# Dnmt2/Trdmt1 as Mediator of RNA Polymerase II Transcriptional Activity in Cardiac Growth

**DOI:** 10.1371/journal.pone.0156953

**Published:** 2016-06-06

**Authors:** Hossein Ghanbarian, Nicole Wagner, Beatrice Polo, Delphine Baudouy, Jafar Kiani, Jean-François Michiels, François Cuzin, Minoo Rassoulzadegan, Kay-Dietrich Wagner

**Affiliations:** 1 Biotechnology Department, School of Advanced Technologies in Medicine, Shahid Beheshti University of Medical Sciences, Tehran, Iran; 2 Institute for Research on Cancer and Aging, Nice (IRCAN), University of Nice Sophia-Antipolis, CNRS UMR7284/INSERM U1081, Faculty of Medicine, Nice, France; 3 Univ. Nice Sophia Antipolis, CNRS, Inserm, iBV, 06100, Nice, France; 4 Department of Pathology, CHU Nice, Nice, France; 5 Department of Molecular Medicine, Faculty of Advanced Technologies in Medicine, Iran University of Medical Sciences, Tehran, Iran; Texas A& M University Health Science Center, UNITED STATES

## Abstract

Dnmt2/Trdmt1 is a methyltransferase, which has been shown to methylate tRNAs. Deficient mutants were reported to exhibit various, seemingly unrelated, defects in development and RNA-mediated epigenetic heredity. Here we report a role in a distinct developmental regulation effected by a noncoding RNA. We show that Dnmt2-deficiency in mice results in cardiac hypertrophy. Echocardiographic measurements revealed that cardiac function is preserved notwithstanding the increased dimensions of the organ due to cardiomyocyte enlargement. Mechanistically, activation of the P-TEFb complex, a critical step for cardiac growth, results from increased dissociation of the negatively regulating Rn7sk non-coding RNA component in Dnmt2-deficient cells. Our data suggest that Dnmt2 plays an unexpected role for regulation of cardiac growth by modulating activity of the P-TEFb complex.

## Introduction

Dnmt2/Trdmt1 is a member of the cytosine-5 methyltransferase family and shows strong sequence conservation to the catalytic motifs of established DNA methyltransferases [1]. However, Dnmt2-dependent DNA methylation has been found to be very low or absent *in vivo* [2], but Dnmt2-dependent methylation of tRNAs, which protects them from cleavage, has been reported [2, 3]. In Zebrafish, Dnmt2 knockdown experiments have been shown to induce lethal differentiation defects in the retina, liver, and brain [4]. Phalke et al. have indicated that Dnmt2 controls transposable elements in Drosophila [5] and Drosophila mutants showed reduced viability under stress conditions [3]. Furthermore, Dnmt2 plays a role in non-random sister chromatid segregation in adult testicular stem cells in Drosophila [6]. Recent studies in mice have shown that RNA- mediated epigenetic heredity requires Dnmt2 [7] and that endochondral ossification is delayed in newborn Dnmt2-deficient mice [8]. Interestingly, Tuorto et al. performed proteomic analyses in this recent study and gene ontology annotation identified cardiovascular disease as the most up-regulated category in Dnmt2-deficient mice [8]. However, the function of Dnmt2 in the heart remained elusive. We report here a cardiac hypertrophy phenotype in Dnmt2-deficient mice, which seems to be governed by noncoding RNAs.

Studies in mouse myocardium have shown that induction of Cdk9/cyclin T1 or Cdk7/cyclin H activity is linked to cardiac hypertrophy [9,10]. Cdk9/cyclin T1 acts as a principal mediator of RNAPII C-terminal domain (CTD) phosphorylation. In particular, RNAPII CTD phosphorylation increases mRNA and protein expression, which mediates cardiac growth [11].

Mouse B2 RNA inhibits RNA polymerase II (Pol II) CTD phosphorylation by TFIIH via interaction with the polymerase [12,13]. *In vitro* studies suggested that the presence of B2 RNA at a promoter prevents phosphorylation of Ser5 residues on the CTD by TFIIH. Sequence analyses of B2 families showed that they are closely related to tRNA genes [14,15].

Rn7sk is a small nuclear RNA that inhibits Cdk9 activity through physical association with the Cdk9/cyclin T complex [16,17]. Rn7sk does not inhibit Cdk9 directly, but bridges P-TEFb (Positive Transcription Elongation Factor b) to Hexim1 (hexamethylene bisacetamide-induced protein 1), which in turn inhibits Cdk9 function [18,19].

We have shown previously that transcriptional induction of Cdk9 following small non-coding RNAs (sncRNAs) injection into one-cell embryos results in cardiac hypertrophy in mice [20]. To investigate a potential role of Dnmt2 for cardiac growth, we examined Cdk9, RNA pol II phosphorylation, and Rn7sk in Dnmt2-deficient mice compared to wild-type littermates. RNA pol II was highly activated in Dnmt2-deficient hearts, which most likely results from a decreased methylation and an increased Rn7sk dissociation from P-TEFb complex. Thus, we postulate that Dnmt2 prevents over-activation of RNA pol II and cardiac hypertrophy.

## Materials and Methods

### Mice and genotyping

**Fig 1:**
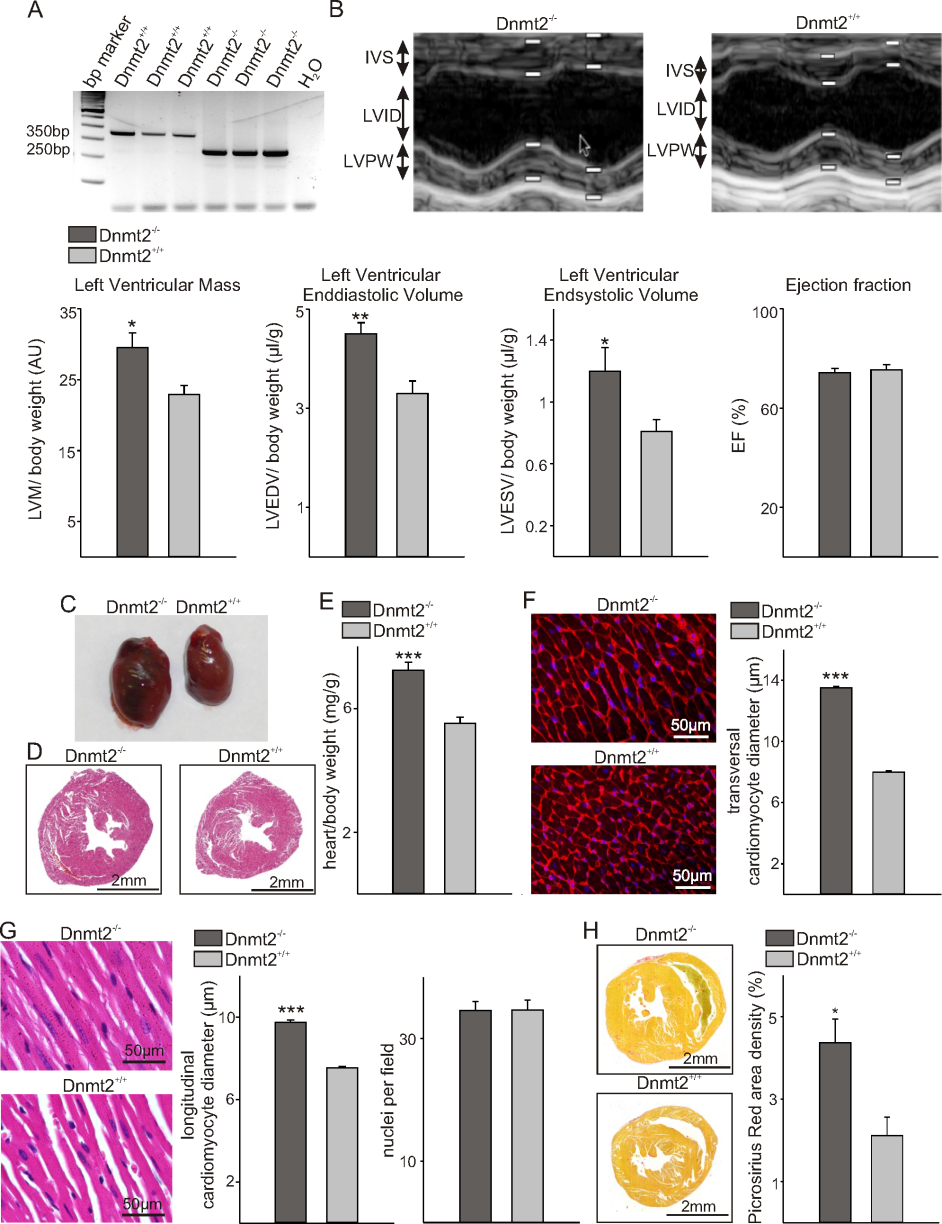
Dnmt2-deficiency induces cardiac growth with preserved function. (A) Representative semiquantitative RT-PCR to genotype Dnmt2^-/-^ and Dnmt2^+/+^ animals. (B) Representative echocardiographic recordings from Dnmt2^-/-^ and Dnmt2^+/+^ animals. The interventricular septum (IVS), left ventricular internal dimensions (LVID), and leftventricular posterior wall (LVPW) are indicated. White bars represent systolic and diastolic measuring points. The arrow represents the cursor position from the echocardiographic recording and has no biological meaning. Lower panel: Echocardiographic examination indicates increased left ventricular mass, systolic and diastolic volumes, but preserved ejection fraction in Dnmt2^-/-^ (*n* = 8) compared to control mice (*n* = 6). (C) Representative photographs of the hearts. (D) Photomicrographs of hematoxylin-eosin (HE) stained cross-sections of the hearts. Scale bars indicate 2mm. (E) Heart-to-body weight ratios (*n* = 8). (F) Photomicrographs of WGA-stained sections showing transversal cross sections of individual cardiomyocytes and quantification of cardiomyocyte diameters (*n* = 5 for each group). (G) High power photomicrographs of HE-stained longitudinal sections showing individual cardiomyocytes and quantification of cardiomyocyte diameters and cellularity per field (*n* = 5 for each group). (H) Representative photographs of Picrosirius Red stained heart cross sections and quantification of Picrosirius Red area densities as a measure for collagen deposits (*n* = 5 for each group). Scale bars indicate 50μm. (F) Data are mean ± SEM. *p < 0.05, **p < 0.01, ***p < 0.001.

The experiments described here were carried out in compliance with the relevant institutional and French animal welfare laws, guidelines and policies. They have been approved by the French ethics committee (Comité Institutionnel d’Ethique Pour l’Animal de Laboratoire; number NCE/2012-54). Dnmt2 -/- homozygote knockout mice [2] were kindly provided by T. Bestor. Originally maintained on a mixed genetic background, the mutation was backcrossed for more than ten generations onto the C57BL/6 genetic background. Genotypes were determined by PCR analysis of Neo and LacZ expression and by Southern blot hybridization using a genomic probe. Routinely, genotyping was performed by PCR yielding bands of 350 bp and 250 bp for the wild-type and knockout allele, respectively (Fig 1A).

### Cardiac experimentation procedures

Three months old male and female mice were used. Xylazine/Ketamine-anaesthetized mice (100 mg/kg body weight for Ketamine and 10 mg/kg body weight for Xylazine injected intraperitoneally) were examined by echocardiography using the iE33 xMATRIX system with a 12MHz transducer (Philips Healthcare, DA Best, Netherlands). Anesthesia lowered the heart rate compared to conscious mice without significant differences for Dnmt2^-/-^ and Dnmt2^+/+^ animals. Histology and measurement of cardiomyocyte diameters was performed according to established protocols [20,21]. Three μm paraffin sections were used for histological and immunohistological procedures. Haematoxylin-Eosin staining was routinely performed on all tissue samples; additionally, sections were stained with wheat germ agglutinin (WGA) (1:100, Life Technologies, Molecular Probes) and Picrosirius Red. Slides were photographed using a slide scanner (Leica Microsystems, Nanterre, France) or an epifluorescence microscope (DMLB, Leica, Germany) connected to a digital camera (Spot RT Slider, Diagnostic Instruments, Scotland). ImageJ was used to determine Picrosirius Red area densities (*n* = 5 animals each), as well as cardiomyocyte diameters at the level of the nucleus in longitudinal sectioned cells of the interventricular septum for the HE staining (*n* = 255 cells for Dnmt2^-/-^ and *n* = 305 cells for Dnmt2^+/+^, *n* = 5 animals each) or cardiomyocyte diameters at the level of the nucleus in transversal sectioned cells of the interventricular septum for the WGA staining (*n* = 391 cells for Dnmt2^-/-^ and *n* = 367 cells for Dnmt2^+/+^, *n* = 5 animals each). Investigators were blinded for the genotype of the mice.

### Cell culture

Mouse embryonic fibroblasts (MEFs) prepared from 12.5 days embryos were inactivated by 10μg/ml mitomycin (Sigma). Dnmt2 knock out, kindly provided by T. Bestor, and control ES cells were grown on inactivated feeder cells in standard ES culture medium. According to well-established procedures [22], embryoid bodies were generated from knock out and control ES cells by culture in hanging drops, the cells were plated back on gelatin-coated plates 3 days later and cardiac differentiation was monitored by the appearance of beating cells.

### Western blot analysis

Total lysates from cell cultures and hearts were prepared, electrophoresed, and blotted. The following antibodies were used for immunodetection: polyclonal anti-CDK9 antibody from rabbit (H-169, sc-8338, Santa Cruz Biotechnology) in a 1:500 dilution in PBS, 2.5% Blotto, 0.05% Tween-20, polyclonal anti-RNA Pol II from rabbit (N-20, Santa Cruz Biotechnology sc-899) 1:500, mouse monoclonal anti ß-actin (Santa Cruz Biotechnology), peroxidase-coupled goat anti-rabbit secondary antibody (Santa Cruz Biotechnology) 1:10000, and peroxidase-coupled rabbit anti-mouse secondary antibody (Santa Cruz Biotechnology) 1:10000. Western Blots (*n* = 2) were quantified using ImageJ with the Gel Analysis tool.

### RNA immunoprecipitation

Total RNA was prepared from hearts of Dnmt2-deficienct mice and wild-type littermates (*n = 6* each) using Trizol Reagent (Invitrogen). Ten μg total RNA were incubated with 2 μg of 5-mC monoclonal antibody (33D3, Diagenode) overnight at 4°C in 500 μl RIP buffer (150 mM KCL, 25 mM Tris pH 7.4, 5 mM EDTA, 0.5 mM DTT, 0.5% NP40, 100 U/ml RNAse inhibitor (Invitrogen)) complemented with protease inhibitor (Complete®, Roche Diagnostics, Indianapolis, USA). Afterwards, protein G beads (50 μl, GE Healthcare) were added and after 1 hour incubation, 3 washes of pelleted beads performed in RIP buffer. 5-mC antibody precipitated RNAs were isolated using Trizol Reagent. cDNA synthesis was performed using the Maxima First strand cDNA Synthesis kit (Thermo Scientific) and quantitative PCR for Rn7sk using SYBR Select Master Mix (Applied Biosystems). Traces of non-specific bound GAPDH served for normalization.

### Northern blot analysis

Northern blot analysis was performed as described [23]. Briefly, 6 μg total RNA extracted from cell cultures and hearts was loaded onto a 12% denaturing polyacrylamide gel and electrophoresed until the bromophenol blue marker reached the bottom of the gel. The separated RNA was electrotransferred to a Hybond N+ membrane (Amersham). Hybridization was carried out in the presence of 32 P-endlabeled DNA oligonucleotide probes. The following probe sequence was used 5’-GAAGAGGACGACCTTCCCCG-3’.

### Quantitative RT-PCR

RNA extraction and quantitative RT-PCR were performed as described [24]. Briefly, RNA was extracted using Trizol Reagent (Invitrogen). 1μg RNA samples were reverse transcribed to cDNA by using random primers hexamers and MLV reverse transcriptase (Invitrogen). Quantitative PCR was performed using the ‘Platinum® SYBR® Green qPCR SuperMix-UDG’ kit (Invitrogen). miR-1 determinations were performed with the Taqman MicroRNA Assay (Applied Biosystems) mmu-miR-1. Sequences of oligonucleotides primers are listed in Table 1. The 2^-ddCT method was used for calculation.

**Table 1:** Primers for quantitative RT-PCR analysis.

Gene	Accession number	Primer symbol	Sequence 5’-3’
Cdk9	NT-033778	Cdk9-F	TAAAGCCAAGCACCGTCAG
		Cdk9-R	GATTTCCCTCAAGGCTGTGAT
myh6	NC_000080.5	myh6-F	CCAAGACTGTCCGGAATGA
		myh6-R	TCCAAAGTGGATCCTGATGA
myh7	NC_000014.8	myh7-F	GGCCTCCATTGATGACTCTG
		myh7-R	CGCCTGTCAGCTTGTAAATG
Gapdh	NM_008084	Gapdh-F	TGTCCGTCGTGGATCTGAC
		Gapdh-R	CCTGCTTCACCACCTTCTTG
Rn7sk	NC_000075.6	Rn7sk-F	AGGACGACCTTCCCCGAATA
		Rn7sk-B	GCGCCTCATTTGGATGTGTC
Ctip2	NC_000078.6	Ctip2-F	TACTGTCACCCACGAAAGGC
		Ctip2-B	TGGGAAGAGGAGGCAGCTAT

### Rn7sk-Cdk9 complex immunoprecipitation

Immunoprecipitation assays were performed as described [25]. ES cells were washed with PBS twice before formaldehyde (1% in PBS) crosslinking. Afterwards, cells were again washed with PBS twice before incubating in lysis buffer consisting of 10 mM HEPES, pH 7.0, 0.5% Nonidet P-40, 100 mM KCl, 5 mM MgCl_2_, 1mM DTT, 100U/ml RNasin (Promega), 2 mM vanadate (Sigma-Aldrich). One tablet of protease inhibitor mixture (Complete Mini®, Roche Diagnostics, Indianapolis, USA) was added just prior to use. Whole cell lysates were incubated on ice for 30 min and the protein concentration was determined using a BCA protein assay kit (Pierce, Rockford, IL). 500 μg proteins were incubated with 10μg polyclonal anti-Cdk9 antibody from rabbit (H-169, sc-8338, Santa Cruz Biotechnology) for 4h. Alternatively, 10 μg of a polyclonal anti-Cyp2 antibody from rabbit (H-21, sc-133491, Santa Cruz Biotechnology) were used as a negative control. Protein A–agarose beads were added and rotated at 4°C for 4h. RNA protein complexes were washed 4 times with polysome lysis buffer (100mM KCl, 5mM MgCl2, 10mM HEPES, 0.5% Nonidet P-40, 1mM DTT, 100U/ml RNasin RNase inhibitor (Promega), 2mM vanadyl ribonucleoside complexes solution (Sigma-Aldrich), 25μl protease inhibitor cocktail (pH 7.0). RNA protein complexes were eluted from beads with polysome lysis buffer including 0.1% SDS and 30μg proteinase K and reverse cross-linked at 50°C for 30 min, followed by TRIzol (Invitrogen) extraction and ethanol precipitation.

### Statistical Analysis

Data are expressed as means ± SEM. ANOVA with the Bonferroni test as post hoc test was used versus controls. Differences between two groups were tested using the Mann-Whitney test for nonparametric samples. Differentiation of 200 wild-type and Dnmt2-deficient ES cell clones each was estimated for each time point using a contingency table with Fisher’s test and Yate’s correction. A p-value less than 0.05 is considered statistically significant.

## Results

### Cardiac hypertrophy in Dnmt2-deficient mice

Echocardiographic examination of adult Dnmt2^-/-^ mice compared to age-matched controls revealed increased left ventricular mass relative to body weight and higher left ventricular end-diastolic and end-systolic volumes whereas the ejection fraction as measure of cardiac contractility was preserved (Fig 1B). Dnmt2-deficient mice showed macroscopically and microscopically increased hearts sizes compared to controls (Fig 1C and 1D). Heart weights relative to body weights were increased on average by 20% (Fig 1E). Body weights were comparable (data not shown). Measurements of cardiomyocyte diameters revealed an increase by approximately 20% in Dnmt2^-/-^ mice compared to wild-type littermates, whereas the cellularity remained unchanged (Fig 1F and 1G). Dnmt2^-/-^ hearts displayed more fibrosis than the wildtype counterparts (Fig 1H).

### Activation of RNA pol II in Dnmt2-deficient cells

**Fig 2:**
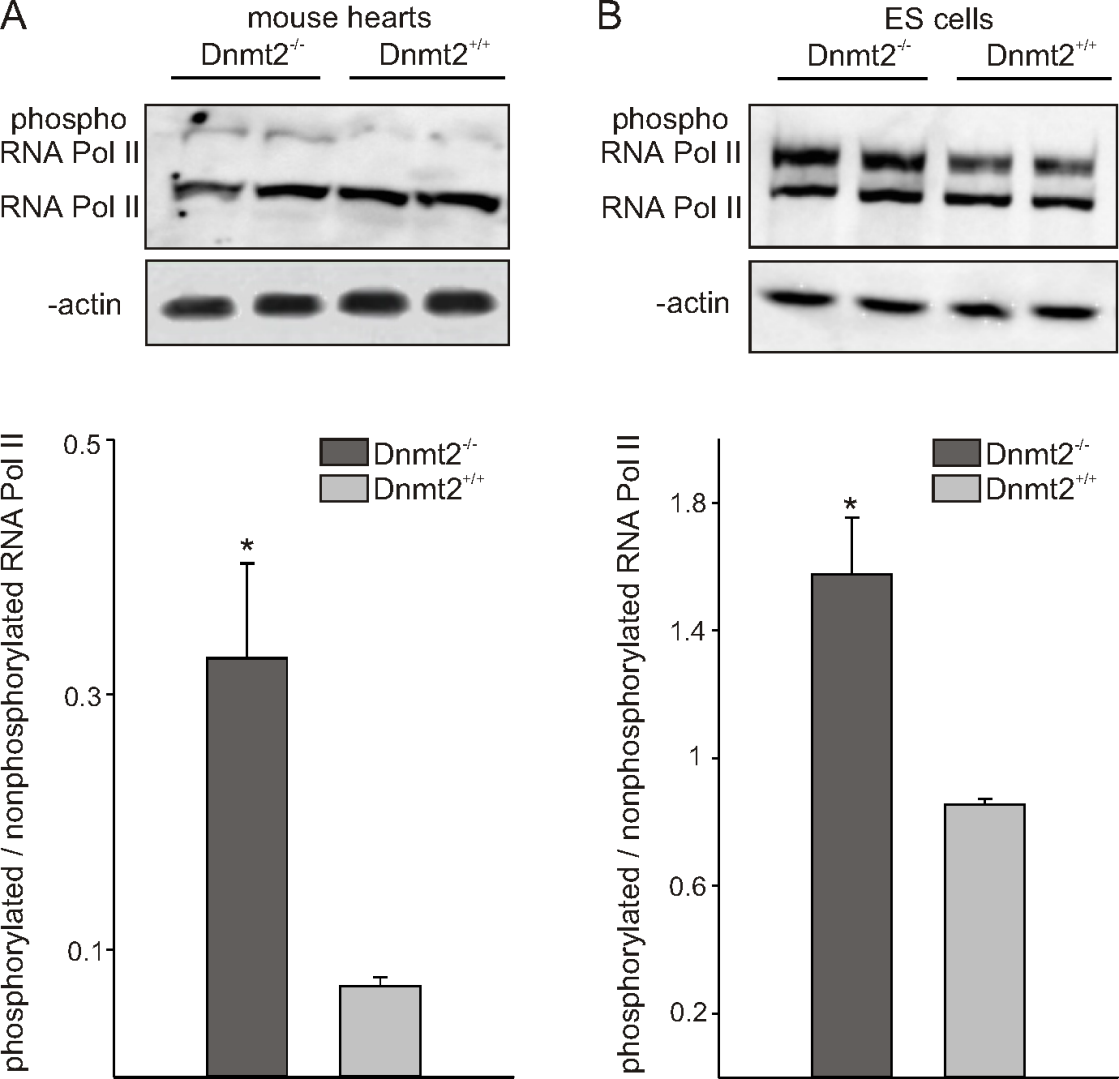
Dnmt2-deficiency is associated with increased phosphorylation of RNA polymerase II. Representative Western Blots for RNA polymerase II (RNA Pol II, upper panels) and quantification of the ratio of phosphorylated to non-phosphorylated RNA Pol II (lower panels) in mouse hearts (A) and ES cells (B) with knockout of Dnmt2 and controls. β-actin was used as a standard. Data are mean ± SEM. *p < 0.05.

To monitor RNA pol II phosphorylation in the heart, we performed Western blotting with an antibody that recognizes both, hyperphosphorylated and hypophosphorylated pol II (IIo and IIa, respectively). Interestingly the proportion of phosphorylated versus total pol II, the form required for productive transcript elongation, increased in Dnmt2-deficient mice (Fig 2A). Similarly, more active form of RNA polymerase II was detectable in Dnmt2-deficient ES cells compared to wild-type cells (Fig 2B).

### Enhanced cardiac differentiation of Dnmt2 knock-out ES cells

**Fig 3:**
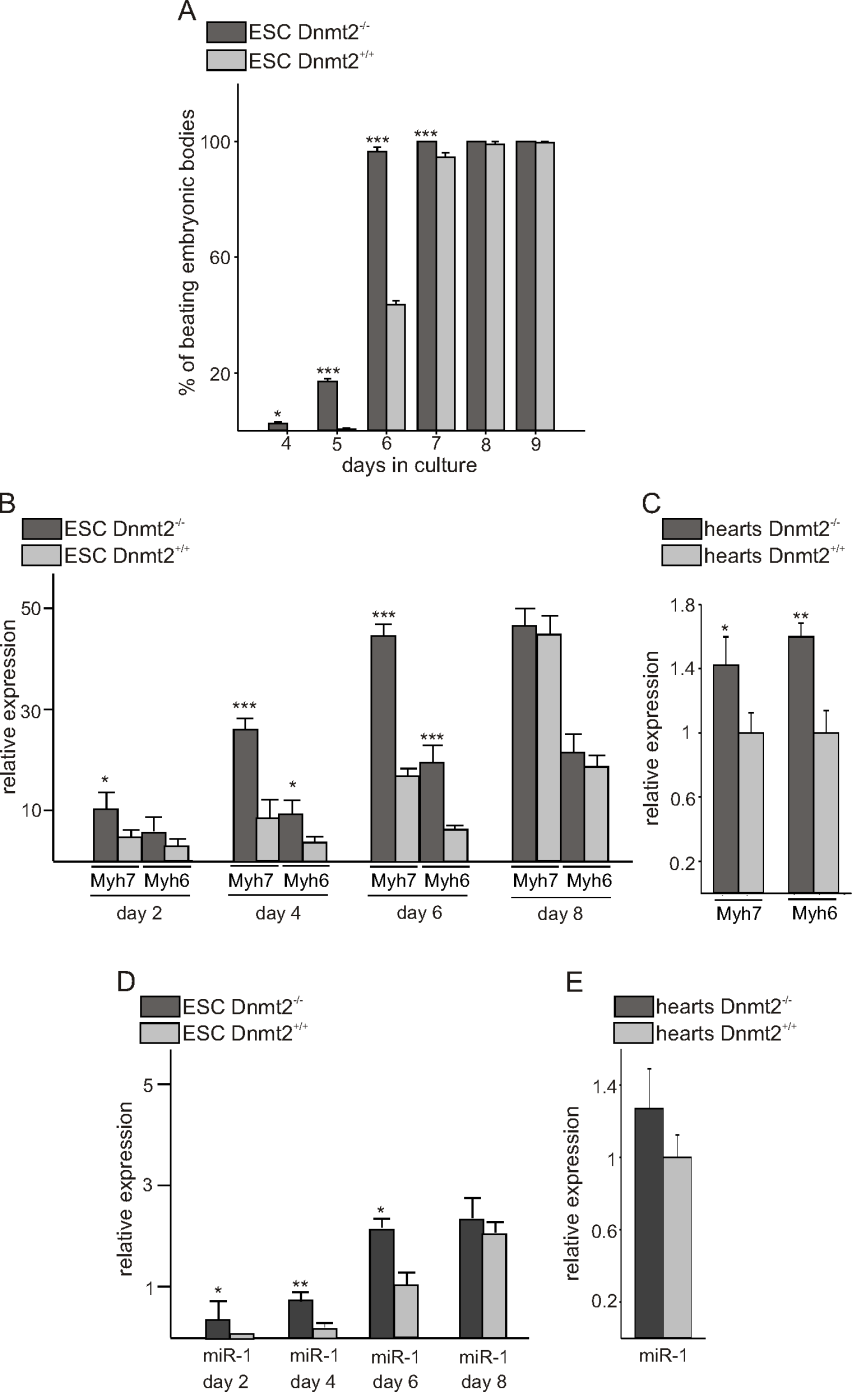
Dnmt2-deficiency enhances cardiac differentiation in ES cells *in vitro*. (A) Embryoid bodies of hanging drop cultures from Dnmt2^+/+^ and Dnmt2^-/-^ ES cells were seated individually (*n = 200* each) in 48-well tissue culture plates and the appearance of beating cells monitored daily. (B) RNA was extracted at different timepoints of differentiation from additional Dnmt2-deficient and control ES cell clones (*n =* 6 each) and quantitative RT-PCR analysis of cardiac differentiation markers Myh6, and Myh7 was performed. (C) Quantitative RT-PCR for Myh6, and Myh7 in Dnmt2^-/-^ and Dnmt2^+/+^ hearts (*n* = 6 for each group). (D) Quantitative RT-PCR for miR1 at different timepoints of differentiation from Dnmt2-deficient and control ES cell clones (*n =* 6 each). (E) qRT-PCR for miR1 in Dnmt2^-/-^ and Dnmt2^+/+^ hearts (*n* = 6 for each group).*p < 0.05, **p < 0.01, ***p < 0.001.

The morphology and growth characteristics of Dnmt2-deficient ES cells were comparable to wild-type ES cells; and they did not exhibit any sign of differentiation, neither into cardiac muscle nor otherwise. As it is established that activated RNA pol II plays a key role in cardiac growth and differentiation and we could detect more active form of RNA polymerase II in Dnmt2-deficient ES cells, we tested the differentiation potency of Dnmt-deficient ES cells *in vitro*. Culture of the Dnmt2-deficient and control ES cells in hanging drops led to their aggregation in embryoid bodies (EB) [22]. After plating the cells back on gelatin-coated plates 2 days later, cardiac differentiation, monitored by the appearance of beating cells, progressed at a faster rate in Dnmt2-deficient cells compare to wild-type ES cells (Fig 3A). While the fraction of beating wild-type EBs was about 50%, it was almost 100% for Dnmt2 Dnmt2-deficient EBs on day 6 of differentiation. Accordingly, quantitative RT-PCR determination of cardiac marker genes showed higher values in Dnmt2-deficient EBs than in wild-type EBs. Myh6, Myh7, and miR-1, which were undetectable in undifferentiated Dnmt2-deficient and wild-type ES cells (data not shown), were found, as expected [26], to be increased along with differentiation (Fig 3B and 3D). Significant up-regulation of Myh6 and Myh7 could also be detected in the hearts of Dnmt2-deficient animals as compared to their wild-type littermates (Fig 3C) whereas miR-1 expression was only slightly increased (Fig 3E).

### The P-TEFb complex is activated in Dnmt2 knock-out cells

**Fig 4:**
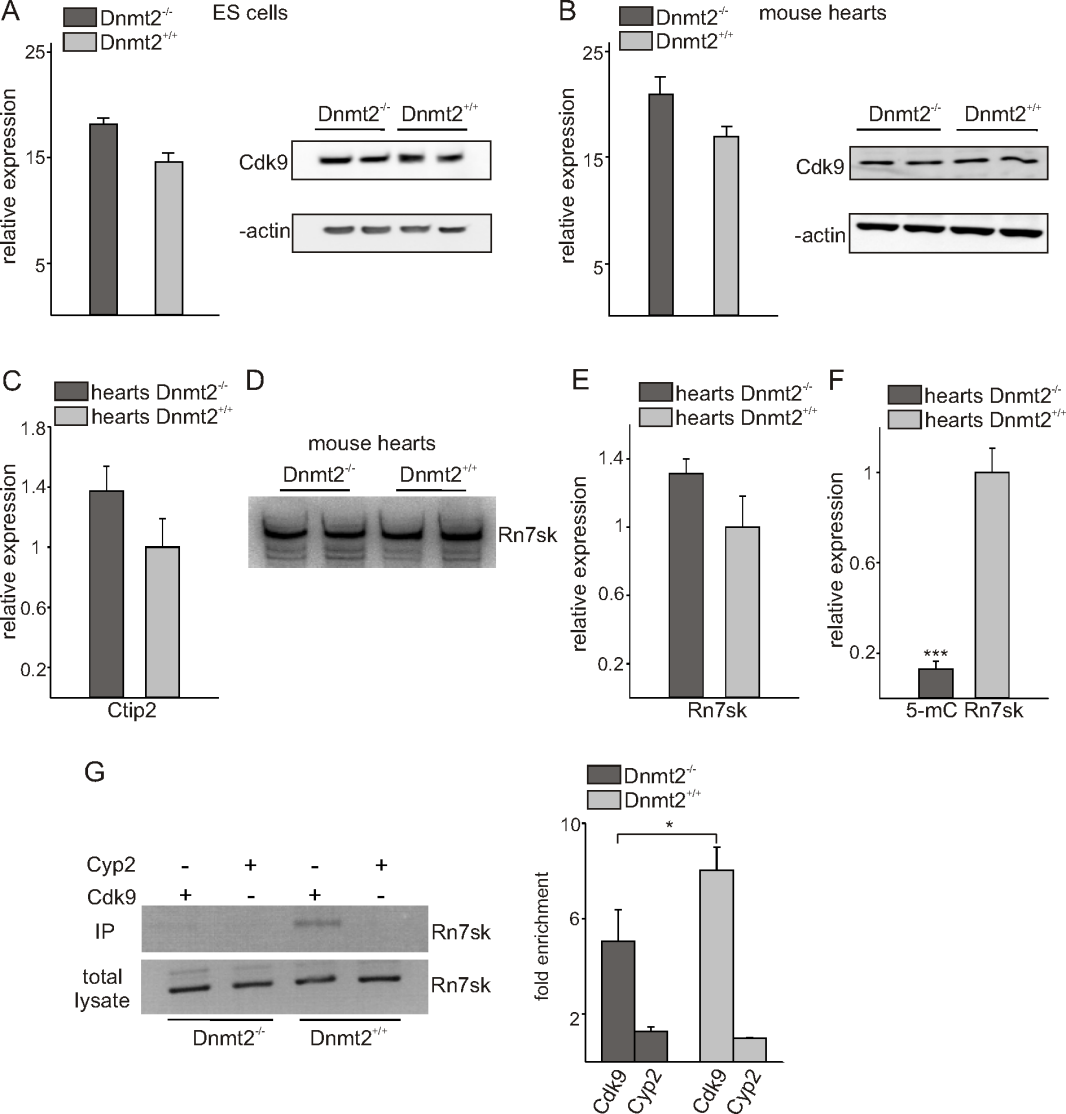
Dnmt2-deficiency affects the P-TEFb complex. (A) Cdk9 expression in Dnmt2-deficient and control ES cells was analyzed by quantitative RT-PCR (left) and Western blot assays (right). (B) Cdk9 expression in Dnmt2-deficient and control hearts was investigated by quantitative RT-PCR (left) (*n* = 5) and Western blot assays (right). (C) Quantitative RT-PCR for Ctip expression in the hearts of Dnmt2^-/-^ and Dnmt2^+/+^ mice (*n* = 6). Northern blot assay (D) and quantitative RT-PCR (E) for Rn7sk (7SK) expression in the hearts of Dnmt2^-/-^ and Dnmt2^+/+^ mice. (F) RNA immunoprecipitation using a 5-methyl Cytidine antibody with RNA extracted from Dnmt2^-/-^ and Dnmt2^+/+^ hearts, followed by RT-PCR for Rn7sk (*n* = 6). Note that Rn7sk is significantly less methylated in Dnmt2^-/-^ hearts. (G) PTEF-b immunoprecipitation using an antibody against Cdk9 on lysates from Dnmt2- mutant and control ES cells followed by RT-PCR for Rn7sk (*n* = 3) and subsequent quantification. An anti Cyp2 antibody served as negative control. Note that less Rn7sk is associated to the P-TEFb complex in Dnmt2-deficient cells.

**Fig 5:**
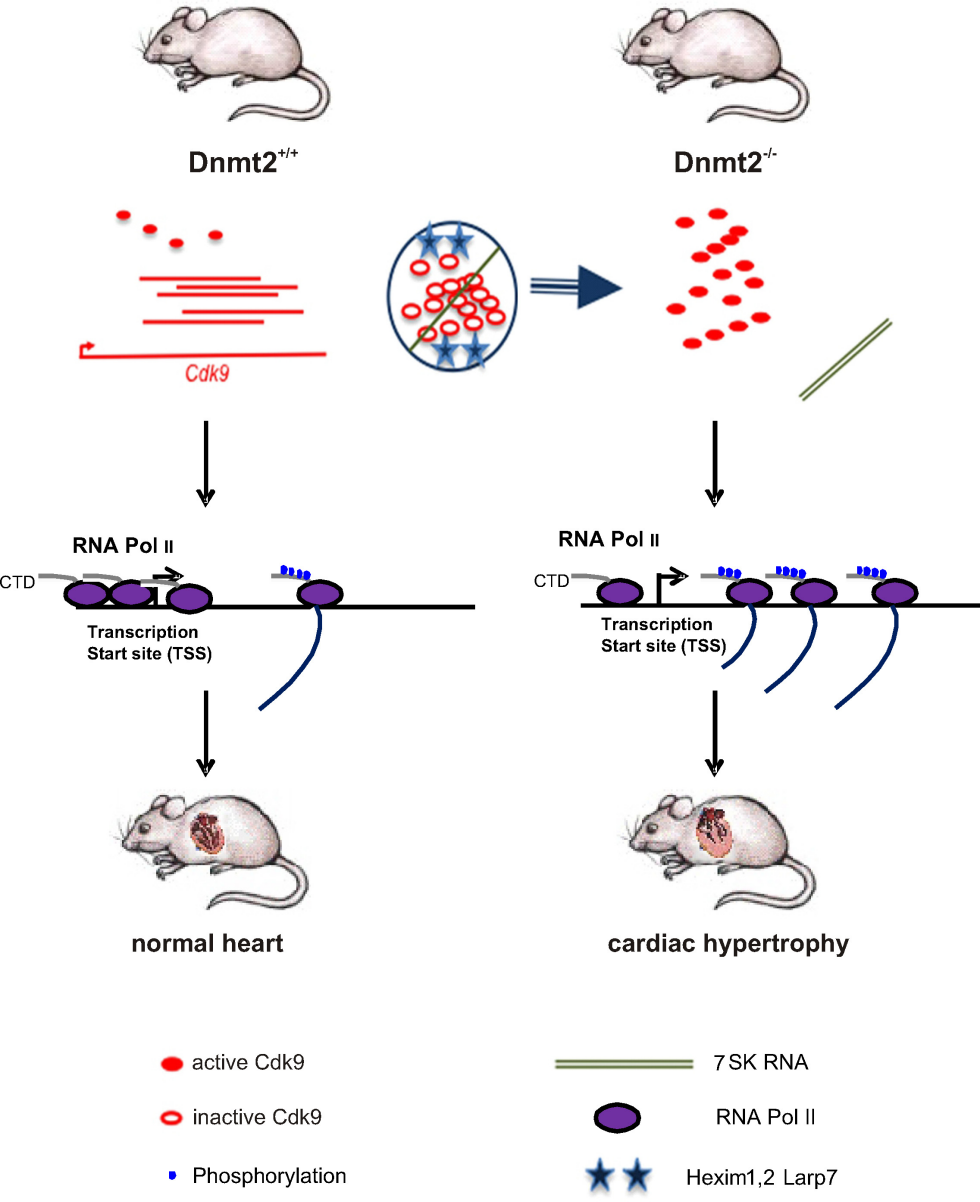
Schematic representation of Dnmt2-mediated RNA polymerase II transcriptional activity in cardiac growth. Storage of inactive protein in a complex with 7SK RNA and inhibitory proteins, release of active kinase (P-TEFb) in Dnmt2 knock out conditions. The phosphorylation of a C-terminal domain of the polymerase by P-TEFb allows elongation of the transcripts. RNAPII CTD phosphorylation increases mRNA and protein expression, which mediates cardiac growth in Dnmt2-deficient mice.

We thought that activation or over-expression of the P-TEFb complex may have a role in the observed phenotype in growth and differentiation of cardiac cells *in vivo* and *in vitro*. Cdk9 positively and Rn7sk negatively regulates the P-TEFb complex, CTIP2 represses the Cdk9 kinase activity of P-TEFb [27]. Cdk9 expression did not differ significantly in Dnmt2- deficient and wild-type ES cells and mouse hearts, neither on the RNA nor on the protein level. (Fig 4A and 4B). Expression of Ctip2 was unchanged in Dnmt2- deficient as compared to wild-type hearts (Fig 4C). Northern blot and RT-PCR assays done with RNA extracted from hearts showed no change in the expression of Rn7sk in Dnmt2-deficient mice compared to controls (Fig 4D and 4E). It has been shown previously that Rn7sk dissociation from the P-TEFb complex is one of the most important P-TEFb activating factors [9–11]. Based on our results we hypothesized that methylation and association of Rn7sk to the P-TEFb complex might have changed in Dnmt2- deficient cells. To test this hypothesis, RNA immunoprecipitation using a 5-methyl Cytidine antibody with RNA extracted from hearts was performed, followed by RT-PCR with specific primers to amplify Rn7sk. The results demonstrate that Rn7sk is significantly less methylated in Dnmt2-deficient cardiac cells (Fig 4F). Additionally, a PTEF-b immunoprecipitation assay was done on lysates from Dnmt2-deficient and wild-type ES cells using an antibody against Cdk9. Immunoprecipitation with an antibody against Cyp2 served as negative control. RNA co-immunoprecipitated by Cdk9 and Cyp2 antibodies was thereafter analyzed by RT-PCR using specific primers to amplify Rn7sk, indicating less enrichment of Rn7sk in Dnmt2-deficient compared to wild-type samples. No enrichment was observed using the Cyp2 antibody indicating specificity for the P-TEFb complex (Fig 4G). A scheme illustrating our findings and hypotheses of Dnmt2-mediated RNA polymerase II transcriptional activity in cardiac growth is provided in Fig 5.

## Discussion

It has been shown that Dnmt2 is part of the RNA processing machinery during cellular stress, by which Dnmt2-mediated tRNA methylation protects tRNA from endonucleolytic cleavage [3]. *In vitro* methylation studies failed to show other substrates for Dnmt2 other than tRNA, which was achieved by incubation of Dnmt2 protein with purified total RNA [2]. It is important to note that these *in vitro* reconstitution studies rely on purified protein components and a controlled environment may not accurately reflect the complex intracellular atmosphere. Besides these *in vitro* studies, and the recent report on the role of Dnmt2 for bone formation in mice [8], little is known about Dnmt2 function in mammals. In the present study, we report a cardiac hypertrophy phenotype in Dnmt2- deficient mice, which seems to be mediated via activation of the P-TEFb complex.

We have previously shown that small non-coding RNAs can induce hereditary epigenetic variations and act as the transgenerational signalling molecules [20, 28, 29]. To consider a role of the Dnmt2 methyltransferase in RNA mediated epigenetic inheritance, we investigated the transgenerational inheritance of previously established phenotypes in Dnmt2 deficient mice [7]. In Kit and Sox9 paramutant models, the epigenetic variants of the respected locus were prevented in Dnmt2- deficient mice [7]. Cdk9 paramutant animals displayed a hereditary cardiac hypertrophy [20]. Thus, we investigated whether Dnmt2 might also be involved in cardiac growth. Interestingly, we describe here a cardiac hypertrophy phenotype in Dnmt2-deficient mice independent from Cdk9 locus induction, mediated by the non-coding RNA Rn7sk. Activation of RNA pol II, a key player in cardiac hypertrophy, was identified not only in Dnmt2- deficient mice, but also in Dnmt2-deficient ES cells. Apart from proteins such as Cdk9, non-coding RNAs including Rn7sk and B2 RNAs tightly regulate RNA pol II activation. We focused on the identification of possible functions of non-coding RNAs in the observed cardiac hypertrophy in Dnmt2- deficient animals. Rn7sk methylation in an NSun2-dependent manner has been already demonstrated [30]. Thus, we focussed on potential methylation of Rn7sk by the other known RNA methyltransferase Dnmt2. Initial bisulfite sequencing was not sensitive enough in our hands to detect robust methylation differences for Rn7sk (data not shown). By RNA immunoprecipitation using a 5-methyl Cytidine antibody followed by quantitative RT-PCR we demonstrate that Rn7sk is significantly less methylated in the Dnmt2^-/-^ than in Dnmt2^+/+^ hearts. This might explain the lack of inhibition of the P-TEFb complex, as RNA methylation protects them from cleavage [2, 3]; and unmethylated Rn7sk might be less stable and more easily degraded. Furthermore, immunoprecipitation assays identified increased Rn7sk dissociation from the P-TEFb complex, which was shown to be important for RNA pol II activation and cardiac hypertrophy [11]. Whether the increased Myh6 and Myh7 expression *in vitro* and *in vivo* results from increased occupancy of the promoters of these genes by the P-TEFb complex or is simply reflecting cardiac hypertrophy remains an open question.

Although we cannot exclude that other yet unidentified mechanisms might contribute to the observed phenotype, the ablation of the Dnmt2 gene is changing the levels of several genes involved in cardiac hypertrophy *in vitro* and *in vivo*. Taken together, we suggest that Dnmt2 is required to limit cardiac growth and differentiation by regulating RNA pol II phosphorylation, which involves the non-coding RNA Rn7sk.
